# Crosstalks of the PTPIP51 interactome revealed in Her2 amplified breast cancer cells by the novel small molecule LDC3/Dynarrestin

**DOI:** 10.1371/journal.pone.0216642

**Published:** 2019-05-10

**Authors:** Eric Dietel, Alexander Brobeil, Lucas Delventhal, Claudia Tag, Stefan Gattenlöhner, Monika Wimmer

**Affiliations:** 1 Institute of Anatomy and Cell Biology, Justus-Liebig-University, Giessen, Germany; 2 Institute of Pathology, Justus-Liebig-University, Giessen, Germany; Duke University School of Medicine, UNITED STATES

## Abstract

LDC3/Dynarrestin, an aminothiazole derivative, is a recently developed small molecule, which binds protein tyrosine phosphatase interacting protein 51 (PTPIP51). PTPIP51 interacts with various proteins regulating different signaling pathways leading to proliferation and migration. Her2 positive breast cancer cells (SKBR3) express high levels of PTPIP51. Therefore, we investigated the effects of LDC3/Dynarrestin on PTPIP51 and its interactome with 12 different proteins of various signal pathways including the interaction with dynein in SKBR3 cells. The localization and semi-quantification of PTPIP51 protein and the Tyr176 phosphorylated PTPIP51 protein were evaluated. Protein-protein-interactions were assessed by Duolink proximity ligation assays. Interactions and the activation of signal transduction hubs were examined with immunoblots. LDC3/Dynarrestin led to an increased PTPIP51 tyrosine 176 phosphorylation status while the overall amount of PTPIP51 remained unaffected. These findings are paralleled by an enhanced interaction of PTPIP51 with its crucial kinase c-Src and a reduced interaction with the counteracting phosphatase PTP1B. Furthermore, the treatment results in a significantly augmented interaction of PTPIP51/14-3-3β and PTPIP51/Raf1, the link to the MAPK pathway. Under the influence of LDC3/Dynarrestin, the activity of the MAPK pathway rose in a concentration-dependent manner as indicated by RTK assays and immunoblots. The novel small molecule stabilizes the RelA/IκB/PTPIP51 interactome and can abolish the effects caused by TNFα stimulation. Moreover, LDC3/Dynarrestin completely blocked the Akt signaling, which is essential for tumor growth. The data were compared to the recently described interactome of PTPIP51 in LDC3/Dynarrestin treated non-cancerous keratinocyte cells (HaCaT). Differences were identified exclusively for the mitochondrial-associated ER-membranes (MAM) interactions and phospho-regulation related interactome of PTPIP51.LDC3/Dynarrestin gives the opportunity/possibility to influence the MAPK signaling, NFkB signaling and probably calcium homeostasis in breast cancer cells by affecting the PTPIP51 interactome.

## Introduction

Breast cancer is the most common invasive cancerous disease amongst women. Prognosis of this disease is greatly influenced whether the Her2-oncogene/oncoprotein is amplified. This applies to 20–30% of the tumors [1]. The amplification of Her2 goes hand in hand with severe alterations in growth and proliferation signaling, e.g., mitogen-activated protein kinase (MAPK) signaling, nuclear factor κ B (NFκB) signaling, by deregulation of signal transduction and protein-protein interactions (PPI) [2]. Detection and understanding of these disturbed signal nodes and PPIs are of the utmost interest in order to develop the most suitable drug for each tumor. Up to now different therapeutic antibodies and tyrosine kinase inhibitors (TKI) like Trastuzumab or Lapatinib have been developed to block the altered Her2 signaling by direct attachment to the Her2 receptor [3]. This targeted therapy led to significantly better results than radio- and chemotherapy alone [4,5]. A drawback to these therapeutics is upcoming resistances of some tumors to the TKIs or the antibody blockage of the receptors [3]. One cause is the early position of the Her2 receptor in the signal transduction which gives the tumor many options to bypass the blocked signaling. In order to overcome such resistance, the identification of drugable PPIs and signal nodes downstream of Her2 is of the utmost interest.

Recently, a novel inhibitor of cytoplasmic dynein, namely LDC3/Dynarrestin was described by Höing et al. [6]. The small molecule interferes with the Hedgehog pathway via inhibition of cytoplasmic Dynein and thereby affecting the intraflagellar transport. A disturbed activation of the Hedgehog pathway is linked to medulloblastoma, basal cell carcinoma, and breast cancer. The scaffolding protein-protein tyrosine phosphatase interacting protein 51 (PTPIP51) was identified as a target of a LDC3/Dynarrestin derived probe in a Yeast-3-Hybrid assay (Lead Discovery Center GmbH, Dortmund, Germany, personal communication). LDC3/Dynarrestin displays PTPIP51 dependent effects on cell signaling, as seen by the knockdown experiments of Brobeil et al. The knockdown of PTPIP51 abolishes the MAPK stimulating effect of LDC3/Dynarrestin normally induced by the PTPIP51/14-3-3/Raf1 interactome [7].

Interestingly, a substrate of the Her2 associated protein tyrosine phosphatase 1B (PTP1B) [8], namely Protein tyrosine phosphatase interacting protein 51 (PTPIP51), couples to the aforementioned MAPK pathway on Raf1 level. PTPIP51 can activate the MAPK pathway by its interaction with 14-3-3β on Raf1 level [9–11]. This leads to enhanced downstream signaling and hence results in cell proliferation, which is a hallmark of malignantly transformed cells, e.g., breast cancer cells. Moreover, PTPIP51 is often deregulated in the development of cancer. In basal cell carcinoma and squamous cell carcinoma, an altered expression is found [12]. Glioblastoma display an increasing PTPIP51 expression, which is associated with higher amounts of 14-3-3β, indicating a higher malignancy [13]. In prostate cancer, the expression of PTPIP51 is enhanced by hypomethylation of its promoter region [14].

Physiologically, PTPIP51 exhibits a heterogeneous panel of functions. PTPIP51 is involved in apoptosis, development, differentiation, cell elongation, migration and NFκB signaling [15–19]. Noteworthy, PTPIP51 as well interacts with VAPB in order to regulate the calcium homeostasis and formation of mitochondria-associated endoplasmic reticulum membranes (MAM) [20,21].

Basing on these facts, we initiated the current study to investigate the modulatory effect of LDC3/Dynarrestin on the Her2 positive breast cancer cell line SKBR3. The modification of PTPIP51 related signaling gives the possibility to influence the associated signaling pathways like the MAPK pathway, NFκB signaling and the calcium homeostasis, which if over-activated or deregulated all lead, to enhanced proliferation, growth, and invasiveness of breast cancer. Thus, PTPIP51 may resemble a new drugable, therapeutic target.

## Materials and methods

### Cell culture

We obtained the SKBR3 cell line from Cell Line Service (Eppelheim, Germany). The cells were cultured in Dulbecco´s MEM (Biochrom) supplemented with 10% fetal calf serum and 1% Penicillin/Streptomycin at 37°C and 5% CO2 in a humidified chamber. The medium was renewed every 2–3 days. They were cultured until 70–80% confluence. Cell harvesting was performed with Accutase treatment for 10 min in a humidified chamber at 37°C and 5% CO2. Subsequently, the cells were rinsed with sterile phosphate buffered saline (PBS) and counted using a Neubauer counting chamber. The cells were seeded in a density of 30.000–40.000 per well in culture slides (Falcon CultureSlides, Corning Life Science, New York, USA, Cat.# 354108).

### LDC3/Dynarrestin

The aminothiazole derivative LDC3/Dynarrestin was synthesized by the Lead Discovery Center, GmbH, Dortmund, Germany. The stock solution in DMSO was stored at -80°C. The synthesis and structural formula of LDC3/Dynarrestin was published by Höing et al. [6].

### Treatment

The cells were allowed to grow for 24h after seeding. Subsequently, they were treated with different concentrations of LDC3/Dynarrestin (diluted in culture medium) for either 1h, 6h, 24h or 48h.

For the activation of NFκB cells were incubated with TNFα (Recombinant Human TNF-α, Peprotech Germany, Hamburg Germany, Cat.# 300-01A) for 6h in a concentration of 100ng/ml. The NFκB inhibition was performed with Ammonium pyrrolidine dithiocarbamate (Sigma-Aldrich, Cat.# P 8765, Munich, Germany) for 6h in a concentration of 50μM. In the case of dual incubation, both compounds were applied at the same time.

The cells were also treated with Sorafenib (LC Laboratories, Woburn, USA, Cat.# S-8599), Dasatinib (LC Laboratories, Woburn, USA, Cat.#. D-3307), 1B Inhibitor (Calbiochem Cat.# 539741) and Ciliobrevin A (Selleckchem, Cat.# S8249).

### Immunocytochemistry

The slides were washed in PBS 2 times for 10 min. The primary antibodies were diluted in blocking solution to the concentration as reported in Table 1. After incubation at room temperature overnight under continuous movement in a humidified chamber, the slides were washed 3 times in PBS for 10 min. The secondary antibodies were diluted (Table 1) in PBS and 10% DAPI was added. The samples were incubated for 45min in a humidified chamber at room temperature in the dark. After washing 3 times in PBS for 10 min, the slides were mounted with Mowiol and stored at 4°C until examination.

**Table 1:** Antibody list.

Name	Immunogen	Antibody source	Clone	Dilution	Manufacturer
**PTPIP51(P51ab)**	Human recombinant PTPIP51 protein encoding amino acids (aa) 131–470	Rabbit polyclonal		1:500	Prof. HW Hofer, Biochemical Department, University Konstanz, Germany
**tyrosine 176 phosphorylated PTPIP51**	Purified total IgG fraction KLH-peptide conjugate	Guinea pig polyclonal		1:400	BioLux, Stuttgart, Germany
**Raf-1**	Mapping the C-terminus of human origin	Mouse monoclonal	E-10	1:100	Santa Cruz Biotechnology Cat.# sc-7267
**14-3-3β**	Specific for an epitope mapping between aa 220–244 at the C-terminus of 14-3-3β of human origin	Mouse monoclonal	A-6	1:100	Santa Cruz Biotechnology Cat.# sc-25276
**PTP1B**	epitope mapping at the N-terminus of PTP1B of human origin	Goat Polyclonal	N-19	1:100	Santa Cruz Biotechnology Cat.# sc-1718
**c-Src**	specific for an epitope mapping between amino acids 1–30 at the N-terminus of c-Src p60 of human origin	Mouse monoclonal	H-12	1:100	Santa Cruz Biotechnology Cat.# sc-5266
**GSK-3β**	raised against amino acids 345–420 mapping at the C-terminus of GSK-3β of human origin	Mouse monoclonal	E-11	1:100	Santa Cruz Biotechnology Cat.# sc-377213
**VAPB**	E.coli-derived recombinant human VAP-B Ala2-Pro132	Mouse monoclonal	736904	1:100	R&D systems Cat.# MAB58551
**Erβ**	raised against ERβ, corresponding to amino acids 256–505 of human origin	Mouse monoclonal	1531	1:100	Santa Cruz Biotechnology Cat.# sc-53494
**Her2**	ERBB2 (NP_004439, 22aa ~ 121aa) partial recombinant protein with GST tag. MW of the GST tag alone is 26 KDa	Mouse monoclonal	22–121	1:100	Abnova, Taipei, Taiwan Cat.# H0000 2064-M05
**RelA**	Recognizes an epitope overlapping the nuclear location signal (NLS) of the p65 subunit of the NFkB heterodimer	Mouse monoclonal	12H11	1:100	Merck Millipore, Schwalbach, Germany Cat.# MAB3026
**IκBα**	Recombinant Human IκB alpha/NFKBIA protein 02	Mouse monoclonal	MM02	1:100	Sino Biological Inc., North Wales, PA, USA Cat.# 12045-H07E
**CGI-99**	epitope mapping at the N-terminus of CGI-99 of human origin	Goat Polyclonal	N-14	1:100	Santa Cruz Biotechnology Cat.# sc-104834
**Nuf-2 (cdcA1)**	raised against amino acids 1–300 mapping at the N-terminus of CdcA1 of human origin	Mouse monoclonal	E-6	1:100	Santa Cruz Biotechnology Cat.# sc-271251
**Phospho-Akt (Ser473)**	a synthetic phosphopeptide corresponding to residues surrounding Ser473 of mouse Akt	Rabbit monoclonal		1:2500	Cell signaling technology #9271
**Phospho-p42/p44 MAPK**	a synthetic phosphopeptide corresponding to residues surrounding Thr202/Tyr204 of human p44 MAP kinase	Rabbit monoclonal		1:2500	Cell signaling technology #9111
**Alexa donkey anti-rabbit Fab fragments**		Donkey polyclonal		1:800	Dianova Cat.# 711-166-152
**Cy3 donkey anti-guinea pig**	IgG (H+L) from guinea pig	Donkey polyclonal		1:800	Dianova Cat.# 706-166-148

### Duolink proximity ligation assay

To determine the interactions of proteins the Olink Duolink Proximity ligation assay (PLA probe anti-rabbit minus, Cat.# 92005, PLA probe anti-mouse plus, Cat.# 92001; Detection Kit Orange, Cat.# 92007) was used. The assay is based on the binding of PLA probes to the primary antibodies. If these are closer than 40nm, a signal is generated. After washing the fixed SKBR3 cells, 10 min in PBS the primary antibodies diluted in blocking solution were applied (concentrations are listed in Table 1). The slides were allowed to incubate overnight in a humidified chamber under continuous movement. The primary antibodies were tapped off, and the slides were washed in PBS 2x10min. PLA probes detecting mouse (Cat# 92001–0100), goat (Cat# 92003–0100) and rabbit antibodies (Cat# 92005–0100) were diluted (1:5) in PBS. Slides were incubated at 37°C in a humidified chamber for 1h. The excess amount of PLA probes was tapped off, and the samples were washed in Wash-Buffer A 2x10min. Duolink II Ligation stock (1:5) and Duolink Ligase (1:40) were diluted in high purity water and added to the slides. After incubation for 30min in a humidified chamber at, 37°C the solution was tapped o, and the slides were washed in Wash-Buffer A 2x5 min. Duolink Polymerase (1:80) and Duolink Amplification and Detection stock (1:5) were diluted in high purity water and added to the samples. The slides were allowed to incubate for 100 min in a humidified chamber at 37°C in the dark. Finally, the slides were washed 2x in Wash-Buffer B for 10 min and 1x in 0,01xWash-Buffer B for 1 min. Nuclear staining was performed using DAPI. After drying for 30 min at room temperature in the dark, they were mounted with Mowiol and stored at 4°C until examination. Leuchowius and coworkers identified the Duolink proximity ligation assay as an adequate tool for the identification of small molecule effectors for protein-protein interactions [22].

### Fluorescence microscopy

The photo documentation was performed with an Axioplan 2 fluorescence microscope equipped with Plan-Apochromat objectives (Carl Zeiss Jena, Jena, Germany). For visualization of the secondary antibody, Alexa Fluor 555 used for the detection of PTPIP51 primary antibody an excitation filter with a spectrum of 530–560 nm and an emission filter with a spectrum 572–647 nm were used. The phosphorylated tyrosine 176 PTPIP51 was visualized by a Cy3 conjugated secondary antibody using the same filter as indicated above.

### Semiquantitative analysis

The immune-cytochemical pictures were analyzed by the ImageJ tool in order to investigate the brightness values. For this purpose cell groups were encircled and analyzed. High staining intensity and therefore high grey value levels display high amounts of protein.

### Protein interaction analysis

For quantification, the DuoLink Image Tool (Olink Bioscience, Uppsala, Sweden, v1.0.1.2) was applied. The software identifies Dapi positive nuclei for the cell count. Cell borders were set according to the software calculated cell shape using a user-defined cell diameter preset. Fluorescence dots of the DPLA were counted by the software for every single marked cell.

### Immunoblot

Samples of SKBR3 cell lysates were separated on Mini-PROTEAN TGX Stain-Free Precast Gels (Bio-Rad, München, Germany Cat.# 4568085). The Bio-Rad Trans-Blot Turbo Transfer System (Bio-Rad, München, Germany) with the settings for mixed molecular weight proteins was used for transfer on an Immobilon P membrane (Millipore, Billerica, USA, Cat.#IPVH07850) according to manufacturer’s instructions. The membrane was blocked with 1x Rotiblock for 1h hour at room temperature. Incubation with anti-pMAPK or anti-Akt was done overnight at 4°C. HRP-conjugated anti-rabbit immunoglobulins diluted in 1x Rotiblock were applied for 1 h at room temperature. The reaction was visualized with the ECL prime substrate. The Bio-Rad ChemiDoc Touch Imaging System (Bio-Rad, München, Germany) was used for documentation. Calibration was performed with a molecular weight marker suitable for chemiluminescence (Life Technologies GmbH, Darmstadt, Germany, Cat.# LC5602). The blots were equalized to the obtained stain-free blot for comparison using the Bio-Rad Image Lab (Bio-Rad, München, Germany). Hence, no loading control is required.

### MTT assay

The cells were seeded in a 96-well plate at a density of 10,000 cells per well and were allowed to grow for 24h. The treatment of the cells was carried out as indicated. The MTT solution was added 4h before the end of the incubation time. After formation of the formazan crystals, the solubilization solution (10% SDS in 0,01M HCl) was added. The solution was carried out overnight in a 37°C 5% CO_2_ humidified chamber. Evaluation of the assay was performed with Berthold Tech TriStar ELISA Reader (Bad Wildbad, Germany).

### Statistical analysis

Data were evaluated using GraphPad Prism 6 software. Statistical significance was determined using ANOVA followed by the Dunnett´s multiple comparison tests. Results were considered significant with p<0.05. (*(p<0.05), **(p<0,01), ***(p<0,001), ****(p<0,0001)). Data in graphs are presented by their mean values and standard deviation.

## Results

### LDC3/Dynarrestin enhanced the Tyr176 phosphorylation of PTPIP51

**Fig 1:**
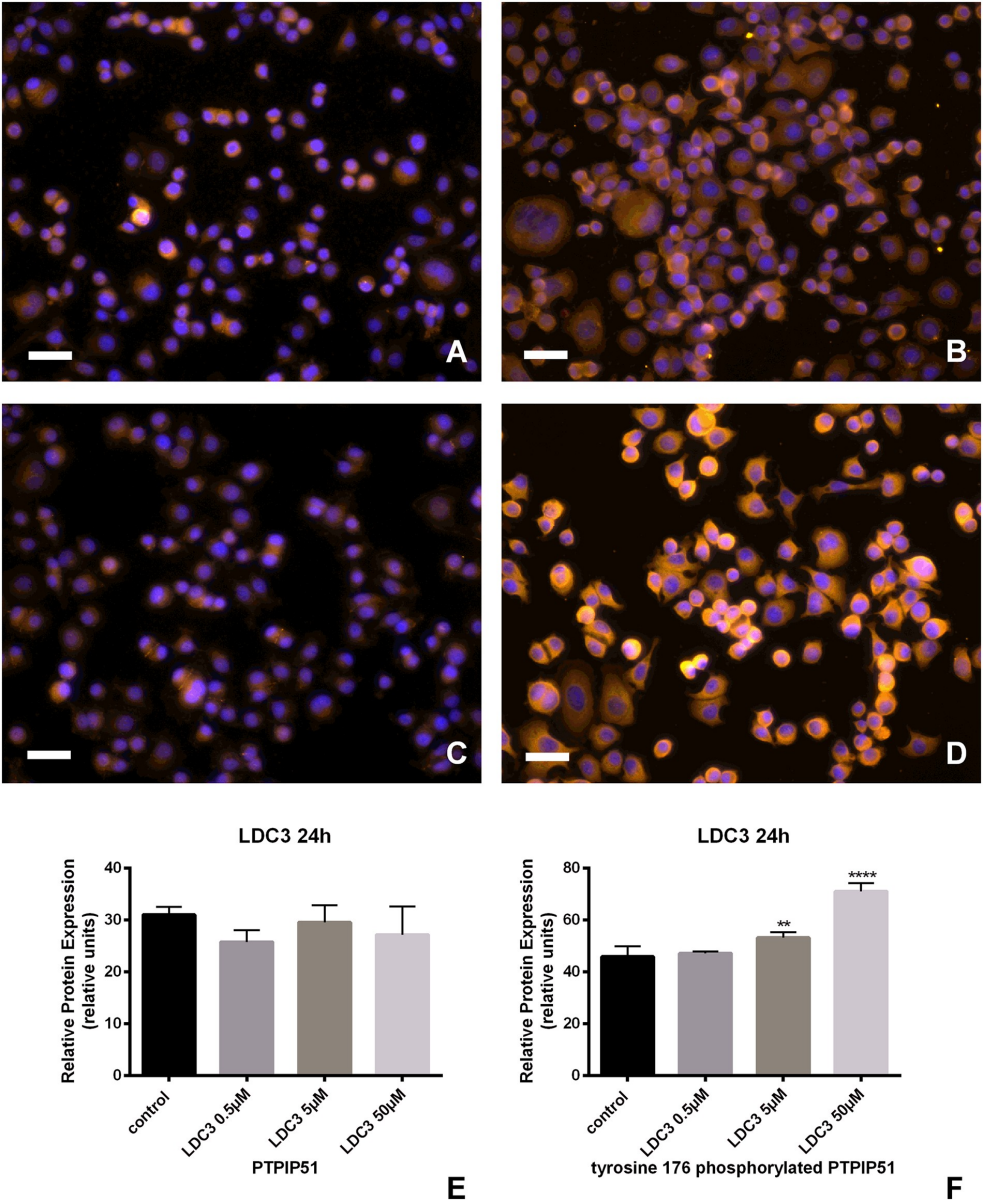
Immunocytochemical staining of PTPIP51 and tyrosine 176 phosphorylated PTPIP51 of SKBR3 cells. **(a)** Immunocytochemical staining of PTPIP51 in untreated cells, **(b)** Immunocytochemical staining of phosphorylated Tyr176 PTPIP51 in untreated cells, **(c)** Immunocytochemical staining of PTPIP51 in 50 μM LDC3/Dynarrestin treated cells (24h), **(d)** Immunocytochemical staining of phosphorylated Tyr176 PTPIP51 in 50 μM LDC3/Dynarrestin treated cells (24h). **(e)** Semiquantitative analysis of the PTPIP51 immunostaining in untreated cell and cell treated with LDC3/Dynarrestin in concentrations of 0.5 μM, 5 μM and 50 μM for 24h, **(f)** Semiquantitative analysis of the phosphorylated Tyr176 PTPIP51 immunostaining in untreated cell and cells treated with LDC3/Dynarrestin in concentrations of 0.5 μM, 5 μM and 50 μM for 24h. Bar = 50μm.

SKBR3 cells either untreated (Fig 1A) or treated with 50 μM LDC3/Dynarrestin for 24h (Fig 1C) were immunostained with antibodies to PTPIP51 and in a parallel experiment to its Tyr176 phosphorylated form (Fig 1B and 1D). The stainings were semi-quantitatively analyzed. The amount of PTPIP51 protein in LDC3/Dynarrestin treated SKBR3 cells was not significantly altered compared to the untreated controls (Fig 1E). In contrary investigating the Tyr176 phosphorylated PTPIP51 in LDC3/Dynarrestin treated cells exhibited a concentration-dependent increase in protein (Fig 1F). The values under 5μM, and 50μM LDC3/Dynarrestin treatment were significantly increased above the control values (p<0.05 and p<0.01, respectively).

### Tyrosine 176 phosphorylation of PTPIP51 under LDC3/Dynarrestin influence in combination with c-Src inhibition and PTP1B inhibition

**Fig 2:**
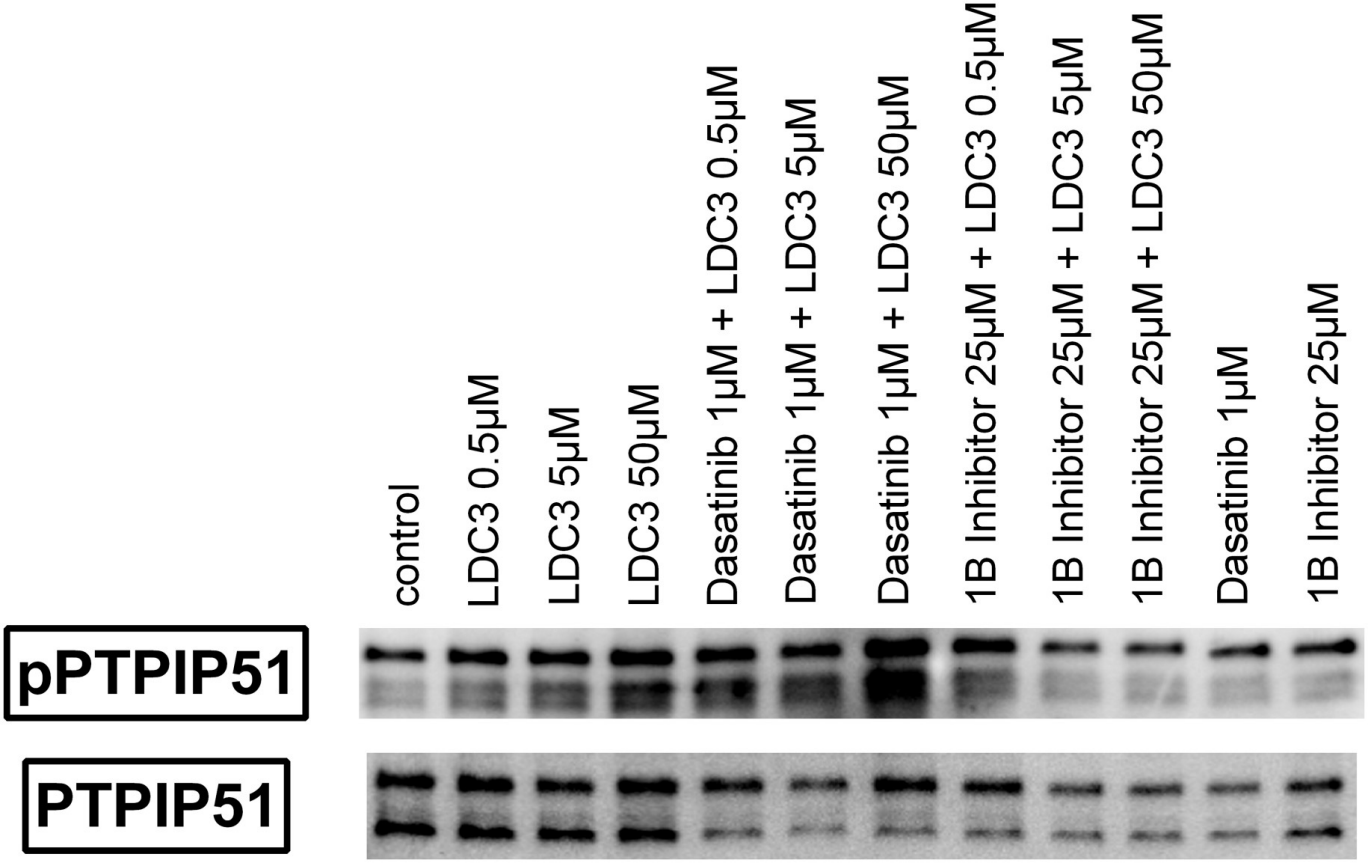
Immunoblots of total PTPIP51protein (PTPIP51) and Tyr176 phosphorylated PTPIP51(pPTPIP51). Cells were treated with LDC3/Dynarrestin, Dasatinib and/or PTP1B inhibitor in the indicated concentration for 1h. Representative blots shown.

The application of LDC3/Dynarrestin in rising concentrations increased the phosphorylation of Tyr176 of PTPIP51 (Fig 2), thus corroborating the findings of the immunocytochemical stainings. The application of the c-Src inhibitor Dasatinib did not interfere with the LDC3/Dynarrestin induced augmentation of Tyr176 phosphorylation of PTPIP51. The combination of rising concentrations of LDC3/Dynarrestin with the PTP1B inhibitor reverted the increase of Tyr176 phosphorylation and induced a reduced Tyr176 phosphorylation of PTPIP51.

### LDC3/Dynarrestin affects the interaction of PTPIP51 and cytoplasmic dynein

**Fig 3:**
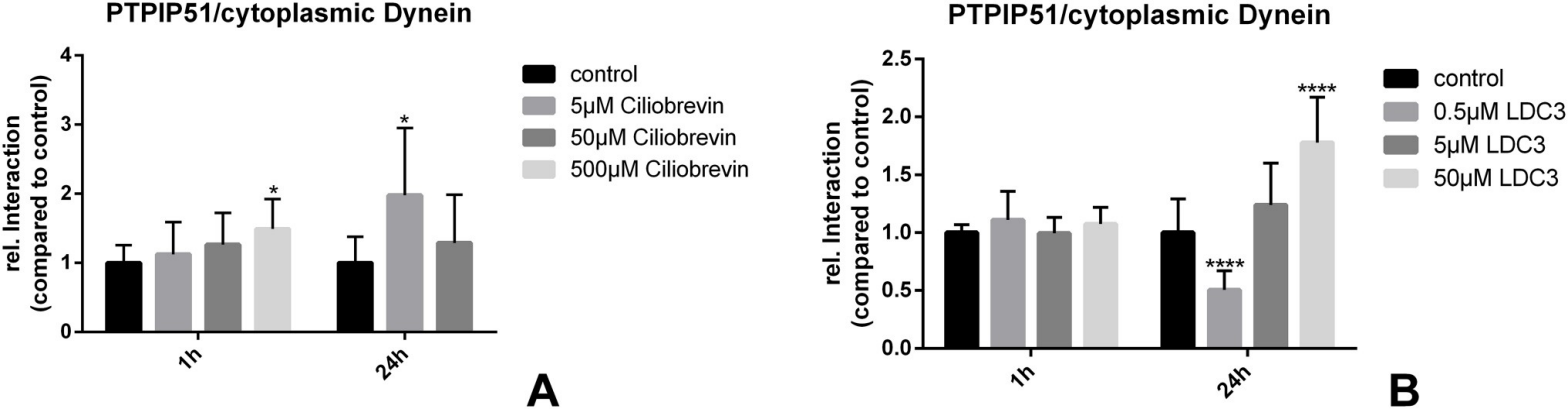
Interactions of PTPIP51 with cytoplasmic dynein in untreated SKBR3 cells and cells treated with LDC3/Dynarrestin (b) or Ciliobrevin A (a) in concentrations ranging from 0.5 μM to 50 μM and 5μM to 500μM, respectively.

LDC3/Dynarrestin inhibits the activity of cytoplasmic Dynein in nanomolar concentrations, through an ATP hydrolysis independent mode of action. Since PTPIP51 is known to interact with cytoplasmic dynein, we monitored the interactional changes of both proteins with proximity ligation assays. If LDC3/Dynarrestin was administrated in nanomolar concentrations (500 nM (p<0.0001)) the interaction of PTPIP51 and cytoplasmic Dynein was reduced if applied for 24h. These effects were reversed if LDC3/Dynarrestin was applied in higher micromolar concentrations (10μM (p<0.0001) and 50μM (p<0.0001)) (Fig 3B). Interestingly, these effects are not seen if LDC3/Dynarrestin was applied for 1h. Application of Ciliobrevin A induced a concentration dependent increase of the PTPIP51/cytoplasmic dynein interaction, but only the highest applied concentration led to a significant increase if applied for 1h. The treatment of SKBR3 cells with Ciliobrevin A for 24h significantly augmented the interaction of PTPIP51 with cytoplasmic dynein, if applied in low concentrations. Of note, the 500μM Ciliobrevin A for 24h severely affected the cell viability, thus preventing a sufficient analysis of protein-protein interactions (Fig 3A).

### LDC3/Dynarrestin affects the MAPK signaling associated interactome of PTPIP51

**Fig 4:**
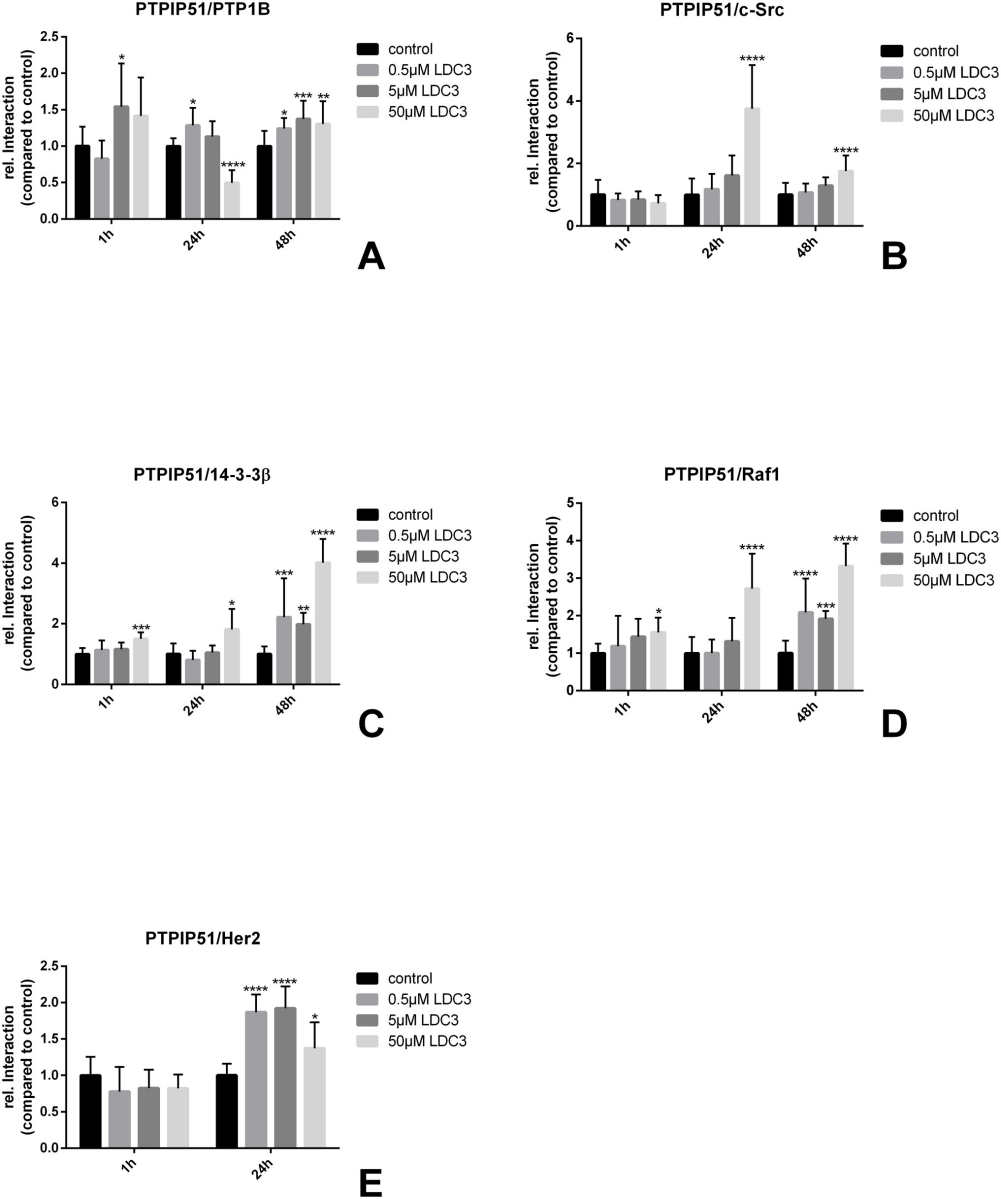
Interactions of PTPIP51 with different partners in untreated SKBR3 cells and cells treated with LDC3/Dynarrestin in concentrations of 0.5 μM, 5 μM, and 50 μM. **(a)** Interaction of PTPIP51 with PTP1B, **(b)** Interaction of PTPIP51 with c-Src, **(c)** Interaction of PTPIP51 with 14.3.3β, **(d)** Interaction of PTPIP51 with Raf1, **(e)** Interaction of PTPIP51 with HER2.

Basing on the observed highly significant alterations in the Tyr176 phosphorylation of PTPIP51, we examined the interaction profile of PTPIP51 with its crucial phosphatase PTP1B. Exposure of SKBR3 cells to LDC3/Dynarrestin led to a triphasic interaction pattern with its tyrosine dephosphorylating enzyme PTP1B (Fig 4A). Application of LDC3/Dynarrestin for 1h at 5μM significantly augmented the interaction of PTPIP51 and PTP1B. If exposed for 24h to increasing LDC3/Dynarrestin concentrations the interaction of PTPIP51/PTP1B was significantly increased (p<0.01) under the lowest concentration of LDC3/Dynarrestin. Application of 5μM LDC3/Dynarrestin resulted in levels higher than normal, whereas 50μM highly significant reduced the number of interactions (p<0.0001). With prolonged incubation times 0.5μM and 5μM significantly surpassed control values (0.5μM p<0.01; 5 μM p<0.001). Although 50μM led to a slight reduction, the values were still significantly higher than those of the controls (p<0.05). Phosphorylation of PTPIP51 at Tyr176 is under control of several tyrosine kinases. One of them is the non-membrane tyrosine kinase c-Src. Interestingly, application of LDC3/Dynarrestin for 1h did not alter the interaction of PTPIP51 and c-Src. The interactions of PTPIP51 and c-Src increased stepwise under LDC3/Dynarrestin treatment both after 24h (5μM p<0.01; 50μM p<0.0001) and 48h incubation time (50μM: p<0.01) (Fig 4B). The observed increase of c-Src/PTPIP51 interaction in combination with the concomitant decrease of PTP1B/PTPIP51 interaction after application of 50μM LDC3/Dynarrestin for 24h explains the increased Tyr176 phosphorylation of PTPIP51. The phosphorylation of Tyr176 is crucial for the interaction of PTPIP51 with 14.3.3β and Raf1, respectively. Treating the cells with LDC3/Dynarrestin changed the interaction of PTPIP51 and 14.3.3β in a concentration and time-dependent manner (Fig 4C). After 24h of exclusive LDC3/Dynarrestin treatment, the highest concentration resulted in a significant increase in the number of interactions (p<0.01). However, doubling the exposure time caused a significant enhancement in interactions independent from the applied LDC3/Dynarrestin concentration in comparison to untreated controls (0.5μM p<0.01, 5μM p<0.0001, 50μM p<0.0001). The interaction profile of PTPIP51/Raf1 (Fig 4D) parallels the one seen for the PTPIP51/14.3.3β interactome. After 24h of LDC3/Dynarrestin treatment, significant increases of the interaction profile were only seen for a concentration of 50μM LDC3/Dynarrestin (p<0.0001). The 48h incubation led to enhanced interactions for all treated cells independent of the applied concentration of LDC3/Dynarrestin. All higher values differed significantly from the controls (0.5μM p<0.001, 5μM p<0.0001, 50μM p<0.0001). Of note, application of 50μM LDC3/Dynarrestin for 1h significantly increased the interaction of PTPIP51/Raf1 and PTPIP51/14.3.3β (Fig 4C and 4D).

Since the amplification of the HER2 receptor in SKBR3 cells directly affects the MAPK signaling, evaluation of the interaction profile of PTPIP51 and HER2 receptor was indispensable. LDC3/Dynarrestin applied for 1h did not lead to a significantly altered interaction of HER2/PTPIP51. These findings correspond with the observations made for the interaction of PTPIP51 and c-Src under the influence of LDC3/Dynarrestin. Treating SKBR3 cells for 24h with LDC3/Dynarrestin resulted in an enhanced interaction of PTPIP51 and HER2 (Fig 4E). Noteworthy, 0.5μM and 5μM LDC3/Dynarrestin led to a highly significant augmentation (p<0.0001), whereas the values are seen after 50μM treatment only differed significantly (p<0.05).

### ERK1/2 and Akt activation under LDC3/Dynarrestin treatment in combination with Raf1 inhibition or c-Src inhibition

**Fig 5:**
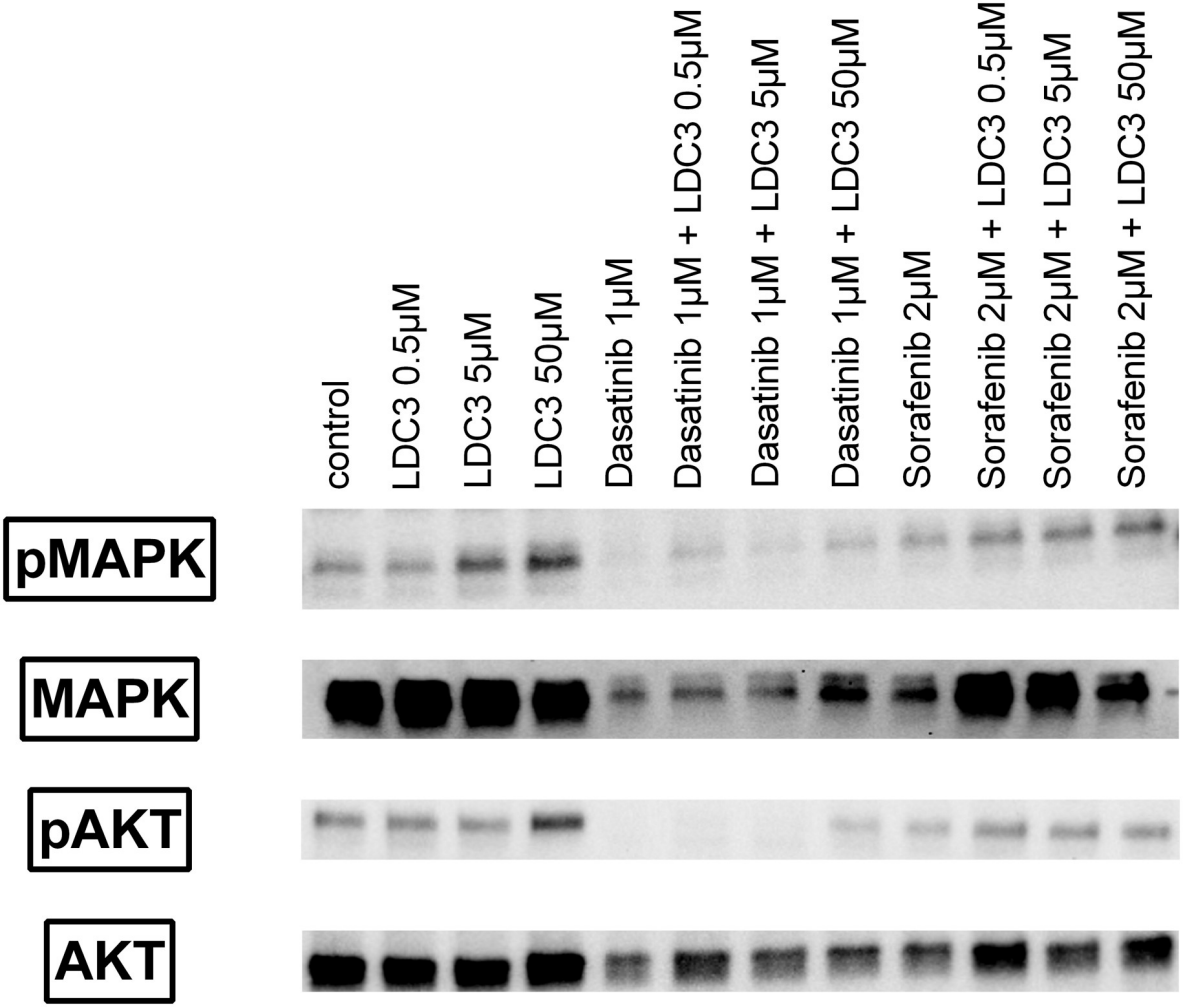
Immunoblots of total ERK1/2 (MAPK), phospho ERK1/2 (pMAPK), panAKT (panAKT) and phospho AKT (pAKT). SKBR3 cell were treated with LDC3/Dynarrestin, Dasatinib and/or Sorafenib in the indicated concentration for 1h. Representative blots shown.

Since LDC3/Dynarrestin enhanced the formation of the Raf1/14.3.3β/PTPIP51 complex, we further examined the influence of LDC3/Dynarrestin on the activation of the MAPK signaling pathway. As an indicator of the MAPK pathway activation the Thr202/Tyr204 phosphorylation of ERK1/2 (pMAPK) was evaluated via immunoblotting. Application of LDC3/Dynarrestin in rising concentrations led to a concentration dependent increase of pMAPK and thus an activation of MAPK signaling. Interestingly, application of the c-Src inhibitor Dasatinib severely reduced the amount of ERK1/2. Nethertheless, the application of LDC3 induced a concentration dependent increase of pMAPK. Likewise, findings were observed for the combination of the Raf1 inhibitor Sorafenib with LDC3/Dynarrestin. The sole inhibition of Raf1via Sorafenib induced an activation of the MAPk pathway compared to the control group. The combination of Raf1 inhibition and low concentrations of LDC3/Dynarrestin slightly reduced the amount of pMAPK. Of note, the combination of Sorafenib with 50μM LDC3/Dynarrestin induced the highest measured amounts of pMAPK (Fig 5).

The Akt signaling represents another central downstream signaling pathway of the HER2 receptor besides the MAPK pathway. In consequence, we also evaluated the phosphorylation of Ser473 of AKT (pAKT) as an indicator of Akt signaling activation. The application of LDC3/Dynarrestin for 1h slightly reduced the amount of pAKT for all applied concentrations. Interestingly, the application of Dasatinib or Sorafenib in combination with LDC3/Dynarrestin reverted the aforementioned reduction of pAkt. The combination of Sorafenib with LDC3/Dynarrestin led to the highest observed pAkt amounts (Fig 5).

### NFκB related PTPIP51 interaction under LDC3/Dynarrestin treatment in combination with stimulation and inhibition of NFκB signaling

**Fig 6:**
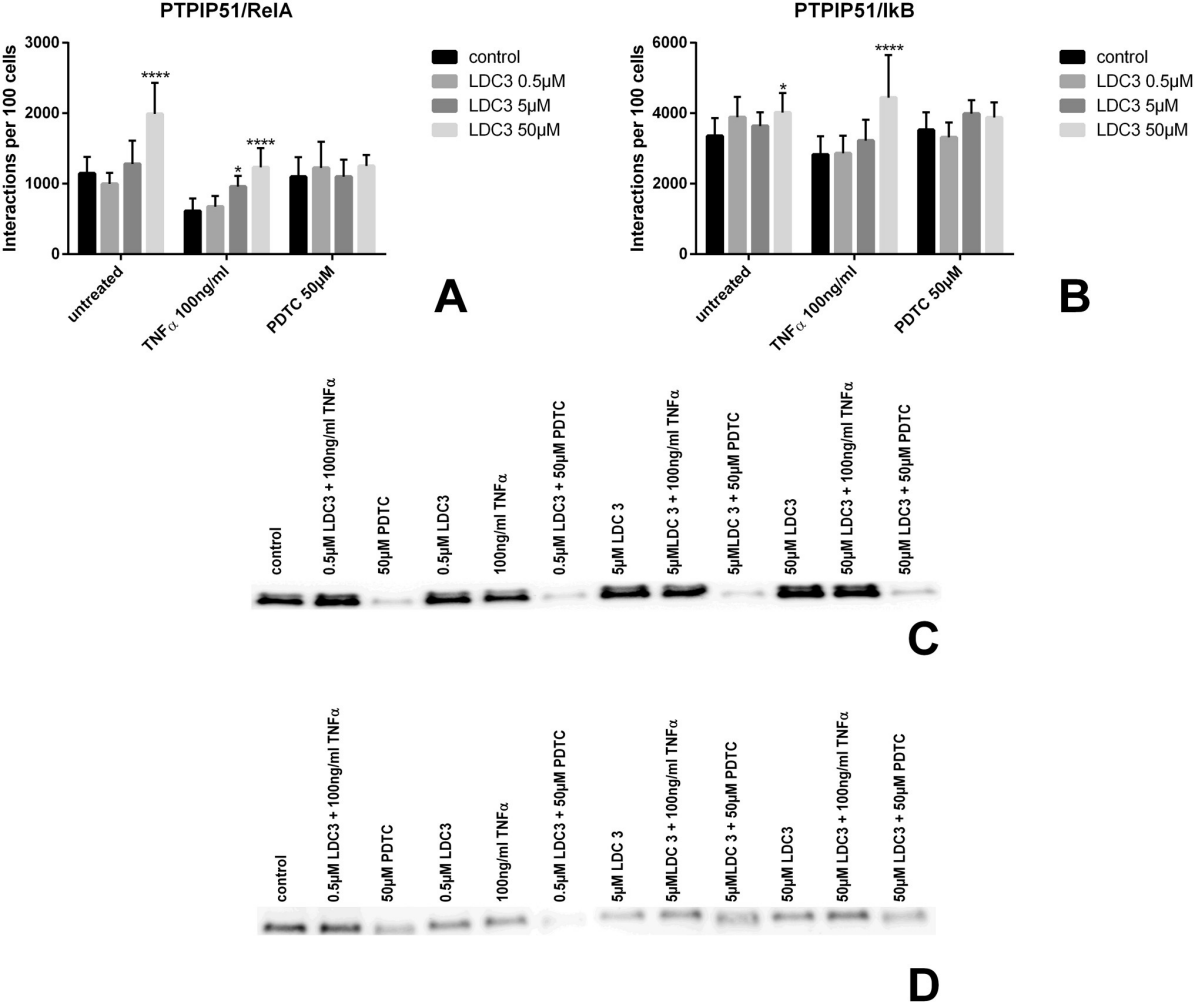
Interactions of PTPIP51 with RelA and IκB in SKBR3 cells either treated with LDC3/Dynarrestin alone or in combination with either TNFα or PDTC in the indicated concentrations for 6h. **(a)** Interaction of PTPIP51 and RelA, **(b)** Interaction of PTPIP51 and IκB. Immunoblots of SKBR3 cells determining the activity of ERK1/2 and Akt by analysing its phosphorylation of specific sites of the molecules. The cells were treated with rising concentrations of LDC3/Dynarrestin. The control groups of the TNFα respectively PDTC subgroup were treated solely with TNFα or PDTC. Incubation time 6h. **(c)** Activation status of ERK1/2, **(d)** Activation status of Akt. Representative blots shown. Normalization was performed using the total protein amount.

The amplification of the HER2 receptor is correlated with an activation of the NFκB signaling. To compare the LDC3/Dynarrestin induced alterations in the NFκB signaling interactome with PTPIP51 in SKBR3 cells with the already well evaluated effects in HaCaT cells, examined by Brobeil and coworkers, we selected an incubation time of 6h [19]. SKBR3 cells exposed to increasing LDC3/Dynarrestin concentrations from 0.5μM up to 50μM displayed an increase in PTPIP51/RelA interactions to about 170% of that seen in untreated cells. Stimulation of NFκB signaling by TNFα (100ng/ml) for 6 h halved the number of PTPIP51/RelA interactions compared to untreated controls. The presence of increasing LDC3/Dynarrestin concentrations led to a stepwise augmentation in the number of interactions near to control values. Inhibition of the NFκB signaling by PDTC treatment (50μM) resulted in the abolishment of the LDC3/Dynarrestin effect (Fig 6A). Comparable observations were made for the interaction profile of PTPIP51 with IκB. The exclusive application of LDC3/Dynarrestin (0.5μM, 5μM, and 50μM) did not increase the number of PTPIP51/IκB interactions (Fig 6B). The simultaneous stimulation of the NFκB signaling pathway by TNFα displayed an insignificant reduction in the number of interactions compared with the untreated control group. The treatment with 50μM LDC3/Dynarrestin and TNFα induced a significant increase of PTPIP51/IκB interactions. PDTC mediated NFκB inhibition resulted in comparable interaction patterns as seen in the untreated controls which correspond to the one observed for PTPIP51/RelA subjected to the same treatments. We also determined the interaction data of PTPIP51 with RelA for a 24h incubation time in order to get better comparability with the interaction profile in HaCaT cells assessed by Brobeil and coworkers [7]. The application of 0.5μM and 5μM LDC3/Dynarrestin highly significant enhanced the interaction of PTPIP51 with RelA (0.5μM p<0.01; 5μM p<0.0001). No significant difference was seen under 50μM LDC3/Dynarrestin treatment compared to the control group (S1 Fig).

To identify cross talks between these signaling hubs and the role of PTPIP51 in their regulation we performed immunoblots of pERK1/2 and pAkt under LDC3/Dynarrestin treatment in combination with NFκB signaling activation and inhibition. The LDC3/Dynarrestin treatment of TNFα stimulated SKBR3 cells for 6h resulted in the same effects on ERK1/2 activation as seen for the treatment with LDC3/Dynarrestin alone. In contrary, the inhibition of the NFκB pathway by PDTC in combination with LDC3/Dynarrestin treatment led to a sharp drop in ERK1/2 phosphorylation and in consequence in its activation (Fig 6C) abolishing the activation enhancing effect of LDC3/Dynarrestin. Akt Ser473 phosphorylation in TNFα stimulated SKBR3 cells was reduced by about 50% compared to the untreated control cells. Combing TNFα stimulation with increasing LDC3/Dynarrestin concentrations resulted in a slight augmentation of the Akt phosphorylation in comparison to solely LDC3/Dynarrestin exposed cells. Noteworthy, PDTC inhibition of NFκB signaling reduced Akt phosphorylation status by about 60%. Combining PDTC and 0.5μM LDC3/Dynarrestin treatment almost abolished Akt phosphorylation, whereas higher LDC3/Dynarrestin concentrations differed only slightly from cells not inhibited by PTDC. Here, PDTC did not affect the LDC3/Dynarrestin effect (Fig 6D).

### LDC3/Dynarrestin induces alterations of the MAM related interactions of PTPIP51 and affects the mitochondrial metabolic rate

**Fig 7:**
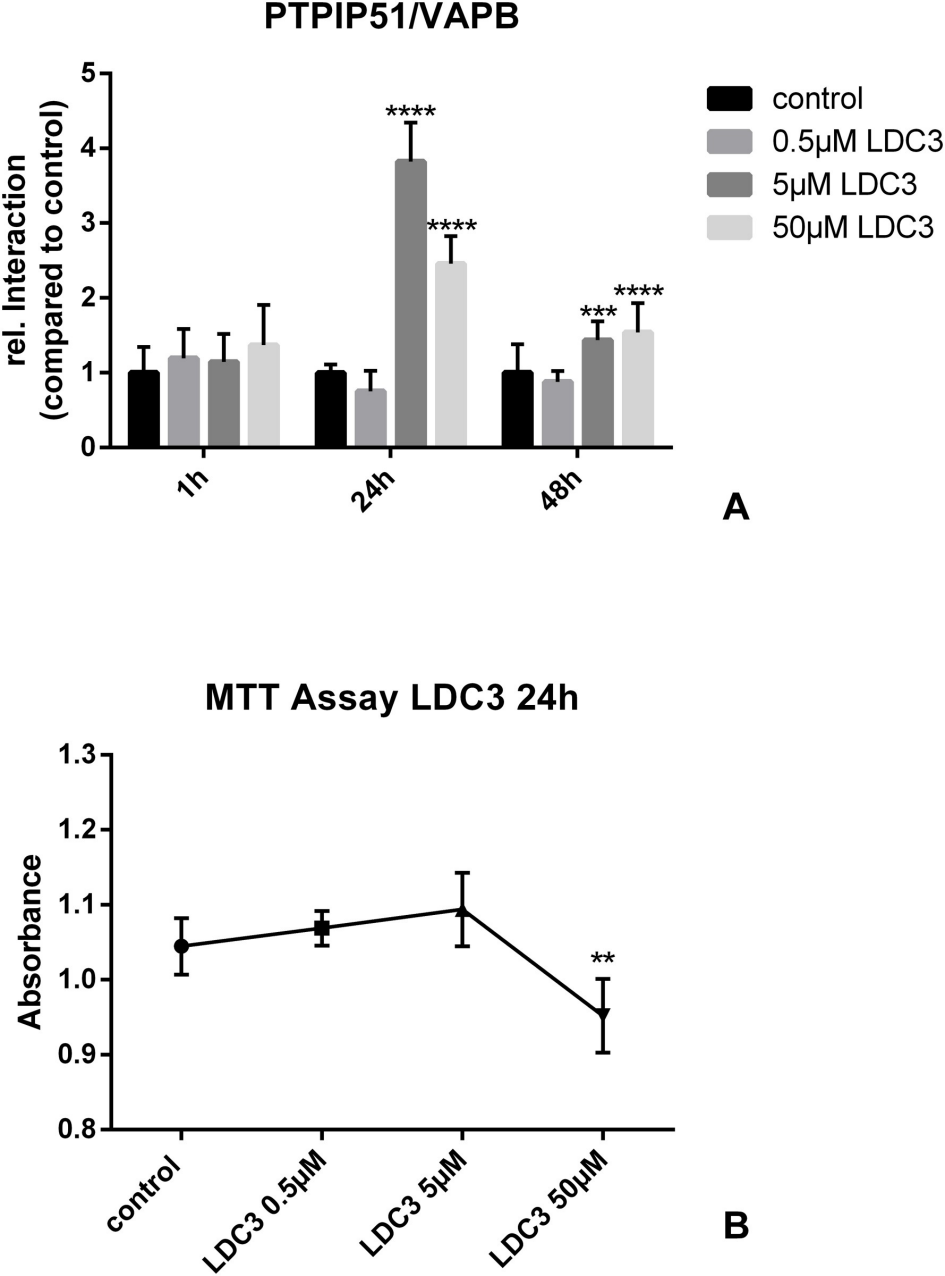
Interactions of PTPIP51 with different partners in untreated SKBR3 cells and cells treated with LDC3/Dynarrestin in concentrations of 0.5 μM, 5 μM, and 50 μM. **(a)** Interaction of PTPIP51 with VAPB, **(b)** Evaluation of the mitochondrial metabolic rate using the (3-(4,5-dimethylthiazol-2-yl)-2,5- diphenyltetrazolium bromide (MTT)) assay. SKBR3 cells were treated for 24h with the indicated concentrations of LDC3/Dynarrestin.

The expression of VAPB in SKBR3 cells was confirmed with an immunocytochemistry (S2 Fig). The interaction PTPIP51 with VAPB was not influenced by low LDC3/Dynarrestin concentrations either after 24h or after 48h of treatment. 5μM and 50μM LDC3/Dynarrestin treatment for 24h induced in cells a highly significant increase by a factor of 3.8 and 2.5, respectively. Such drastic differences were no longer seen after 48h LDC3/Dynarrestin exposure. Nevertheless, the values differed significantly from that measured in untreated controls (5μM p<0.05, 50μM p<0.05) (Fig 7A).

LDC3/Dynarrestin significantly reduced the mitochondrial metabolic rate as assessed by MTT assay if 50μM were applied (p<0.01). The treatment of SKBR3 cells with 0.5μM or 5μM LDC3/Dynarrestin did not alter the mitochondrial metabolic rate (Fig 7B).

### Mitosis-associated interaction of PTPIP51 remained unaffected by LDC3/Dynarrestin treatment

LDC3/Dynarrestin treatment was unable to affect the interaction profiles of PTPIP51 with two proteins involved in mitosis, namely CGI-99 and Nuf2, which are acknowledged interaction partners of PTPIP51 [23]. Neither the interaction of PTPIP51 and CGI-99 nor the interaction of PTPIP51 and Nuf2 displayed any significant variations after 24h of treatment with concentrations of 0.5μM up to 50μM LDC3/Dynarrestin treatment (S3A and S3B Fig).

### Interaction of PTPIP51 with ERβ

If incubated for 24h with LDC3/Dynarrestin the number of PTPIP51/ERβ interactions was significantly increased (S3C Fig). After an application of LDC3/Dynarrestin for 48h, no changes in the interactions at low concentrations (0.5μM and 5 μM) were observed. 50μM LDC3/Dynarrestin led to a highly significant decrease in the number of PTPIP51/ERβ interactions compared to the untreated controls (p<0.0001).

## Discussion

Modulating the PTPIP51 interactome in a HER2 amplified breast cancer cell line (SKBR3) with the novel small molecule LDC3/Dynarrestin led to significant alterations of several tumor relevant pathways. We specifically selected the HER2 positive SKBR3 cell line due to the alterations in signaling caused by the amplified HER2 receptor. HER2 affects the MAPK pathway, Akt signaling, NFκB signaling and calcium homeostasis [24–26]. All these different signaling pathways contain signaling proteins which interact with PTPIP51 [9,19–21,23]. Therefore, the SKBR3 cell line represented a perfect model for the investigation of the LDC3/Dynarrestin affected PTPIP51 interactome. Previous work by Brobeil and coworkers characterized the interaction profile of PTPIP51 under LDC3/Dynarrestin treatment in the spontaneously immortalized HaCaT cell line, which represents a physiological model of cell signaling [7]. The comparison of the PTPIP51 interactome variations in the tumor cell line SKBR3 and the non-tumor cell line HaCaT allows a deeper insight into the dysregulated signaling structures in cancer cells.

LDC3/Dynarrestin inhibits cytoplasmic dynein activity in an ATP hydrolysis independent mode of action [6]. Brobeil and coworkers also showed that LDC3/Dynarrestin could directly affect the PTPIP51 related interactome in HaCat cells. Here, LDC3/Dynarrestin exerts its effects through a PTPIP51 dependent modulation of interactions, as shown by PTPIP51 knockdown experiments [7]. Since LDC3/Dynarrestin affects cytoplasmic Dynein and PTPIP51, we monitored the interaction of cytoplasmic Dynein and PTPIP51 via proximity ligation assay. LDC3/Dynarrestin affects the interaction in a dose-dependent manner. Nanomolar concentrations of LDC3/Dynarrestin reduced the interaction of cytoplasmic dynein and PTPIP51, whereas micromolar concentrations induced an enhancement of the interaction. Such biphasic regulations are already known for PTPIP51. Roger et al. described a biphasic expression peak during the treatment of rat retinal explants with ciliary neurotrophic factor [27]. Of note, these interaction changes were only seen after 24h incubation time. When incubated for 1h, LDC3/Dynarrestin did not significantly alter the interaction of PTPIP51 and cytoplasmic Dynein, implying that the regulation of the PTPIP51/cytoplasmic Dynein interaction could be due to secondary mechanisms. Whereas, the application of Ciliobrevin A, an ATP hydrolysis dependent cytoplasmic Dynein inhibitor, immediately exerts an effect on the interaction of PTPIP51 and cytoplasmic Dynein, when applied in high concentrations.

The regulation of PTPIP51 interactions is mediated through the modulation of its phosphorylation [9]. One of the main regulatory phosphorylation sites of PTPIP51 is the tyrosine 176 residue. Its phosphorylation annuls the ability of PTPIP51 to bind to Raf1 through 14.3.3 and thereby its MAPK pathway stimulating effect. LDC3/Dynarrestin treatment of SKBR3 cells led to a high Tyr176 phosphorylation level of PTPIP51. The same effects were observed in the HaCaT cell line.

Despite the augmented phosphorylation level of the Tyr176 residue, we observed recruitment of PTPIP51 into the MAPK signaling as indicated by the enhanced interaction of PTPIP51 with Raf1 and 14.3.3, respectively. Thus, LDC3/Dynarrestin abolishes the known phospho-regulations of PTPIP51 protein-protein-interactions and forces PTPIP51 into MAPK signaling regardless of its phosphorylation. These observations are paralleled by the MAPK signaling activation under LDC3/Dynarrestin treatment. The findings are in accordance with the data obtained in the HaCaT cell line. Thus, the LDC3/Dynarrestin altered the phosphorylation of the Tyr176 residue and the recruitment of PTPIP51 into MAPK signaling are based on the same modulations both in the breast cancer cell as well as in the physiological system of the HaCaT cell.

Interestingly, inhibition of c-Src or Raf1, two upstream modulators of the MAPK signaling, in combination with low concentrations of LDC3/Dynarrestin reduced the activation of the MAPK signaling compared to control groups. This reduction of MAPK activation is reverted by high concentrations of LDC3/Dynarrestin implying, that LDC3/Dynarrestin is able to activate the MAPK signaling independently of c-Src and Raf1.

The phosphorylation of PTPIP51 is precisely regulated by different kinases and phosphatases. One of the Tyr176 phosphorylating kinases is the non-membrane tyrosine kinase c-Src. C-Src plays a crucial role in the formation of therapy resistance in HER2 amplified breast cancer. Interestingly, LDC3/Dynarrestin enhanced the phosphorylation of Tyr176 of PTPIP51 even under inhibition of c-Src.

PTP1B is mainly responsible for PTPIP51 dephosphorylation. PTP1B is a known positive modulator of the HER2 receptor. Therefore, both PTPIP51 regulating enzymes are potential tumorigenic factors, which presumably exert their effects through modulation of PTPIP51. This thesis is supported by the fact that in LDC3/Dynarrestin treated SKBR3 cells the interaction of PTPIP51 with c-Src and PTP1B is regulated exactly in an opposite way compared to the regulation in HaCaT cells. Interestingly, the inhibition of PTP1B induced results completely opposite of the known regulation. The inhibition of PTP1B in combination with the application with rising concentrations of LDC3/Dynarrestin reduced the phosphorylation of Tyr176 of PTPIP51. This further supports the thesis of a complete disruption of the known phospho-regulation of PTPIP51 by LDC3/Dynarrestin.

Since both PTPIP51 phosphorylation regulating enzymes also modulate the HER2 receptor, we also monitored the interaction of PTPIP51 and HER2. Of note, the application of LDC3/Dynarrestin did not alter the interaction of PTPIP51 and HER2, when applied for 1h. After 24h LDC3/Dynarrestin enhanced the interaction of PTPIP51 and the HER2 receptor. In accordance to the observed interaction changes of PTPIP51/c-Src and PTPIP51/cytoplasmic Dynein, this regulation could be due to secondary mechanisms [28].

TNFα, an activator of the transcription regulator NFκB is highly expressed in breast carcinomas [29]. Moreover, TNFα leads to an augmented expression of prooncogenic factors correlating with poor clinical outcome [30]. Recently, PTPIP51 was identified as a novel partner in NFkB signaling [19]. Strikingly, lower LDC3/Dynarrestin concentrations significantly augmented the interaction of PTPIP51/RelA. The TNFα treatment of SKBR3 cells led to the dissolution of the RelA/IκB/PTPIP51 complex. This effect was already stated for HaCaT cells [19]. The simultaneous application of LDC3/Dynarrestin in rising concentrations completely abolished the TNFα effect. Noteworthy PDTC, an NFκB inhibitor, did not alter the interactions of PTPIP51 neither with RelA nor with IκB but canceled the LDC3/Dynarrestin effect [31]. These results strongly favor the thesis that LDC3/Dynarrestin does not directly influence the RelA/IκB/PTPIP51 interactome but inhibits the activation on the level of IKKs or even upstream. A potential mechanism for the IKK inhibition could be the inhibition of Akt by LDC3/Dynarrestin. Our results showed an inhibition of Akt signaling after 1h and 6h incubation time. Akt is an IKK activator [32]. Therefore, the Akt inhibition might lead to a reduced IKK activity, which ultimately allows the RelA/IκB/PTPIP51 complex to stabilize.

Interestingly, in the SKBR3 cells, LDC3/Dynarrestin induced the same enhancement of PTPIP51/RelA interaction as seen in the HaCaT cells. LDC3/Dynarrestin altered the regulation of the Tyr176 residue phosphorylation, the interaction with MAPK signaling and the link to NFκB signaling via RelA in breast cancer cells and in non-tumor HaCat cells all analogously. This might be a hint for the absence of a dysregulated PTPIP51 regulation in this pathway within the breast cancer cell line.

Besides the MAPK pathway and the NFκB signaling, PTPIP51 is also involved in the formation of the MAMs via the interaction with VAPB. VAPB is highly expressed in many breast tumors [33]. Our HER2 overexpressing model cell line (SKBR3) also displayed high expression of VAPB (S2 Fig). The increased interaction of PTPIP51 and VAPB presumably leads to a more stabilized endoplasmatic reticulum-mitochondria association [20,21]. As mentioned above for several interactions, this regulation is only seen for longer incubation times, implying that the enhanced interaction of PTPIP51 and VAPB might be caused by secondary mechanisms and not directly by LDC3/Dynarrestin. The enhanced interaction of PTPIP51 and VAPB might be a counterregulation to the mitochondrial metabolic stress induced by high concentrations of LDC3/Dynarrestin, but further studies would be needed to proof this thesis.

Interestingly, all the PTPIP51/VAPB interactions are is exactly altered contrary to that seen in HaCaT cells. This may indicate a modified regulation of the MAM associated interactome of PTPIP51 in HER2 positive breast cancer cells. MAM related PTPIP51 interactome in neuronal cancer cells seem to underlie similar mechanisms as seen in preliminary results of an ongoing study. The regulation of these organelle communication sites is of utmost interest since recent findings indicate a crucial role of the MAMs in the regulation of tumorigenesis and tumor cell growth [25].

## Conclusions

**Fig 8:**
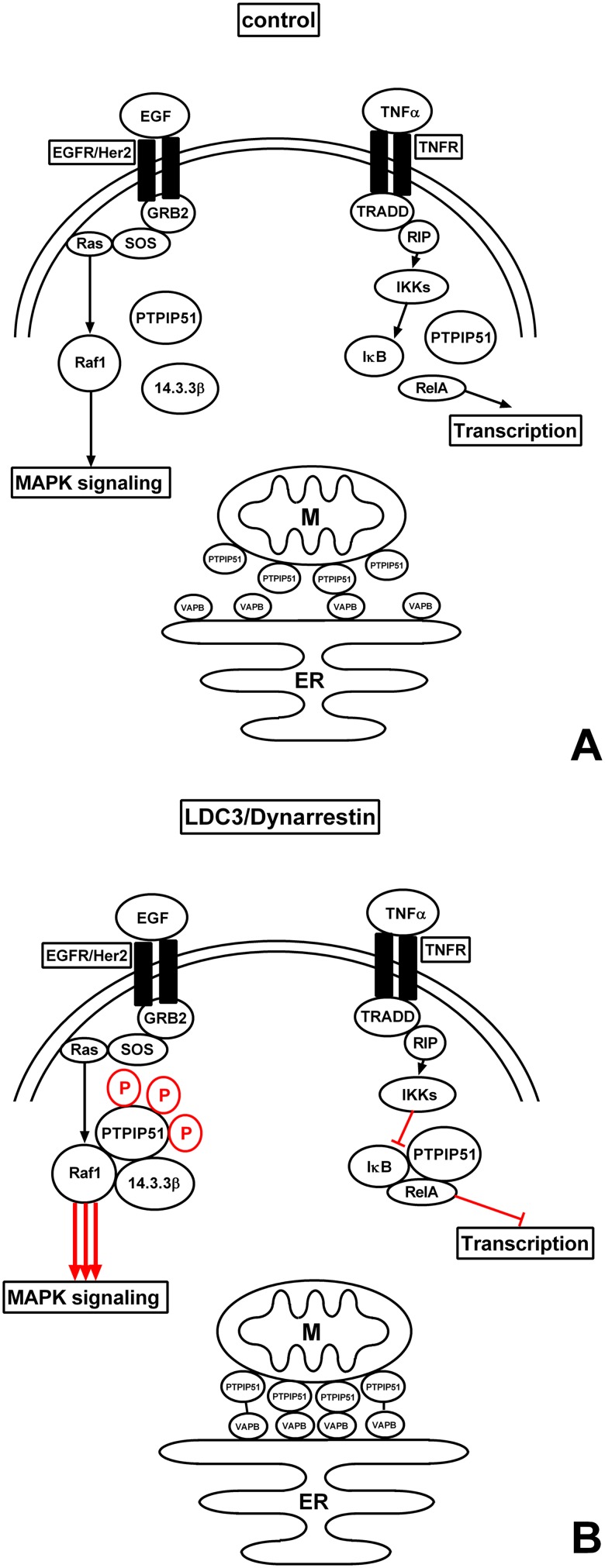
Schematic overview of main signaling pathways and their crosstalks. **(a)** Signaling without LDC3/Dynarrestin, **(b)** Signaling under the influence of LDC3/Dynarrestin.

LDC3/Dynarrestin induces alterations in all known PTPIP51 related signaling pathways. (1) LDC3/Dynarrestin enhances the phosphorylation of Tyr176 of PTPIP51. (2) Despite the high Tyr176 phosphorylation, PTPIP51 is forced into the Raf1/14.3.3β/PTPIP51 complex by LDC3/Dynarrestin. (3) LDC3/Dynarrestin leads to an activation of the MAPK pathway independent of c-Src and Raf1 activity. (4) LDC3/Dynarrestin modulates the NFκB related interactions of PTPIP51. (5) LDC3/Dynarrestin affects the MAM-related interaction of PTPIP51 and VAPB (Fig 8). In conclusion, LDC3/Dynarrestin represents a valuable tool compound for the modulation of PTPIP51 related signaling pathways in physiological and pathologic signaling systems.

## Supporting information

S1 Fig Interaction of PTPIP51 with RelA. Cells were treated in the indicated concentrations of LDC3/Dynarrestin for 1h and 24h.
(TIF)

S2 Fig Immunocytochemical staining of VAPB of SKBR3 cells. **(a)** VAPB distribution in untreated SKBR3 cells, **(b)** negative control. Bar = 50μm.
(TIF)

S3 Fig Interactions of PTPIP51 with different partners in untreated SKBR3 cells and cells treated with LDC3/Dynarrestin in concentrations of 0.5 μM, 5 μM, and 50 μM. (a) Interaction of PTPIP51 with CGI-99, (b) Interaction of PTPIP51 with NUF-2, (c) Interaction of PTPIP51 with ERβ.
(TIF)
