# Supplementary figures and images for: Crosstalks of the PTPIP51 interactome revealed in Her2 amplified breast cancer cells by the novel small molecule LDC3/Dynarrestin
Source: PLoS One. 2019 May 10;14(5):e0216642. doi: 10.1371/journal.pone.0216642 (PMC6510450; doi:10.1371/journal.pone.0216642)

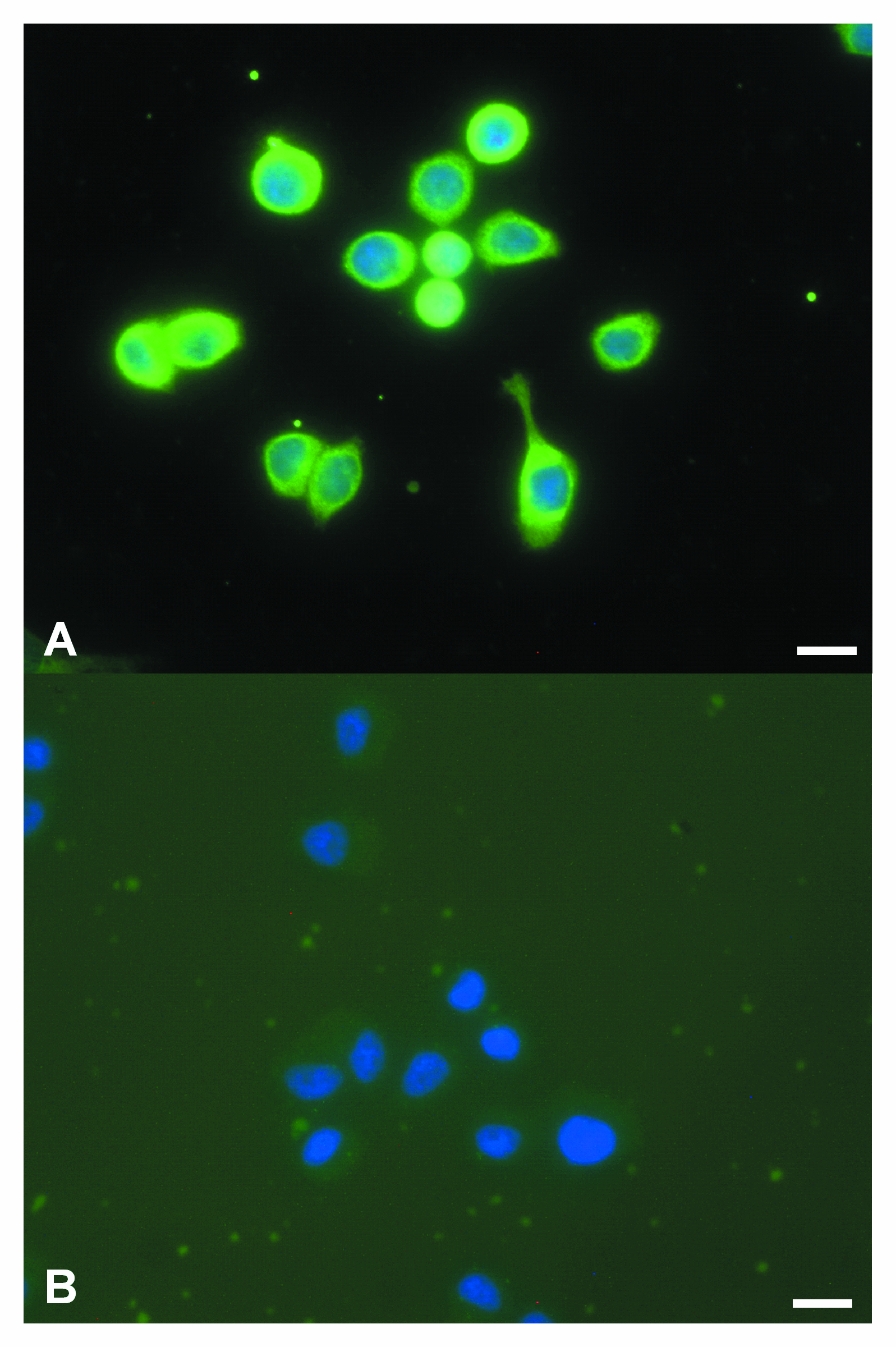

Supplement: S2 Fig — (a) VAPB distribution in untreated SKBR3 cells, (b) negative control. Bar = 50μm. (TIF) [file pone.0216642.s002.tif]
